# Controlled Dispersion and Transmission-Absorption of Optical Energy through Scaled Metallic Plate Structures

**DOI:** 10.3390/ma16186146

**Published:** 2023-09-10

**Authors:** Hammou Oubeniz, Abdelhaq Belkacem, Hicham Mangach, Muamer Kadic, Abdenbi Bouzid, Younes Achaoui

**Affiliations:** 1 Laboratory of Optics, Information Processing, Mechanics, Energetics and Electronics, Department of Physics, Moulay Ismail University, Zitoune, Meknes B.P. 11201, Morocco; h.oubeniz@edu.umi.ac.ma (H.O.); a.bouzid@umi.ac.ma (A.B.); y.achaoui@umi.ac.ma (Y.A.); 2 Light, Nanomaterials Nanotechnologies (L2n), CNRS-ERL 7004, Université de Technologie de Troyes, 10000 Troyes, France; 3 Institut FEMTO-ST, UMR 6174, CNRS, Université de Franche-Comté, 25000 Besançon, France

**Keywords:** metallic array, subwavelength, plasmonic structure

## Abstract

The dispersive feature of metals at higher frequencies has opened up a plethora of applications in plasmonics. Besides, Extraordinary Optical Transmission (EOT) reported by Ebbesen et al. in the late 90’s has sparked particular interest among the scientific community through the unprecedented and singular way to steer and enhance optical energies. The purpose of the present paper is to shed light on the effect of the scaling parameter over the whole structure, to cover the range from the near-infrared to the visible, on the transmission and the absorption properties. We further bring specific attention to the dispersive properties, easily extractable from the resonance frequency of the drilled tiny slits within the structure. A perfect matching between the analytical Rigorous Coupled Wave Analysis (RCWA), and the numerical Finite Elements Method (FEM) to describe the underlying mechanisms is obtained.

## 1. Introduction

The investigation of electromagnetic wave behavior with sub-wavelength metallic structures represents a highly dynamic and productive field of scientific research. It holds significant potential of applications in various areas, including optoelectronics, biosensors, photodetectors, focusing techniques, and plasmonic lithography [1,2,3,4,5,6,7]. The analysis of electromagnetic transmission phenomena through apertures can be traced back to the pioneering contributions of Rayleigh, Wood, Sommerfeld, and Bethe [8,9]. Of particular significance, Bethe’s theory initiated the characteristics of a sub-wavelength circular aperture in an infinitely thin, perfectly conducting plate, through simplified assumptions [8]. In 1998, Ebbesen’s et al. seminal work on extraordinary light transmission through a lattice of sub-wavelength holes within a genuine metallic plate gained considerable interest. Notably, the observed ability to transmit a substantial quantity of light is larger than predicted by Bethe’s theory [10,11]. These investigations have explored the interplay between various types of resonance, including the surface plasmon resonance occurring at the metal/dielectric interface [12], the Fabry-Perot resonance within the aperture [13], and the propagated and/or evanescent modes within waveguides [14]. Subsequently, a wide range of configurations has been extensively investigated, encompassing individual holes or slots [15,16,17], holes encircled by etchings [18], and arrays of slots and holes [19,20,21,22,23].

Besides, the interaction between light and a metallic slot grating exhibits a pronounced dependence on the polarization of the incident wave [24]. In the context of rectangular slots, the optical response of the system displays a polarization-dependent cut-off frequency, notably for the transverse electric (TE) and transverse magnetic (TM) polarizations. This arises from the necessity of maintaining continuity for the parallel component of the electric field at the interface between the metal and air. When the sub-wavelength slit extends infinitely in one direction, the TE mode, characterized by an electric field vector parallel to the long axis of the slit, is unable to transmit through the structure if the incident light’s wavelength exceeds twice the width of the slit [25]. Conversely, the TM mode, exhibits an electric field perpendicular to the long axis of the slit (referred to as the TEM mode), undergoes transmission without encountering any cut-off frequency [26]. However, in the case of real metals, the determination of a precise cut-off wavelength becomes challenging due to the gradual transition from the propagation regime to the evanescence one [27].

In order to gain insights into the underlying physical mechanisms governing light transmission through a metallic array of sub-wavelength nano-slits, various theoretical and experimental investigations have been conducted. These endeavors have significantly improved our understanding of the electromagnetic characteristics exhibited by these slits across different frequency ranges. These ranges span from the ideal regime; perfect metal, where the slit is treated as a waveguide between two perfectly conducting walls, to the absorptive regime; lossy metal. Moreover, extensive studies have been conducted on the impact of shape and size on the transmittance of individual holes. In this context, Garcia-Vidal et al. empirically established a strong association between the transmittance of a rectangular aperture and the width-to-length ratio of the rectangle, thereby establishing that this ratio may serve as a governing factor for the transmission peak [28].

An experimental investigation involving silver (Ag) and gold (Au) films presented a demonstration of the impact of geometrical parameters on the transmission characteristics of sub-wavelength slits [29]. The findings reveal that increasing the slit width results in an increase in transmission, while an intriguing observation of damped oscillation behavior in transmission was noted by increasing the plate thickness. In a separate study, Fernández-Domínguez et al. delved into the influence of the number of slots within a one-dimensional perfect metallic lattice on transmission resonances [21]. The paper highlights the sensitivity of surface modes to this parameter, shedding light on its significance. Besides, Takakura et al. demonstrated that a periodic grating composed of slits displayS the same resonance peaks as a single slit, but with a sharp enhancement of amplitude. This phenomenon transforms efficiently the grating into an amplifier of these resonances [30].

In the perfect regime, the sub-wavelength metallic grating exhibits characteristics comparable to a dielectric layer, with a frequency-independent effective refractive index controlled by the geometrical parameters, as demonstrated in [31]. Bravo-Abad et al. have stated that this transmission is closely associated with the excitation of the waveguide resonances and further established its dependence on the angle of incidence [16]. Additionally, studies by Hsieh et al. [32] and Porto et al. [33] have presented a multi-band transmission achieved through the coupling of incident TEM modes to the guided modes. The coupling is accomplished through the excitation of surface modes, which enables an alternative mode of transmission. The latter, the excitation of surface plasmon polaritons coupled on both surfaces of the metal grating [33].

S. Astilean et al. established a connection between the transmission enhancement and the presence of Fabry-Perot resonances within the open cavities formed by the slits, which involved surface plasmons along the walls of the metal slit [13]. Fuzi Yang et al. experimentally confirmed the Fabry-Perot-like behavior, with slight deviations in the resonance peaks compared to those predicted by the conventional Fabry-Perot model [12]. Consequently, a transmission enhancement via the excitation of resonant waveguide modes within cavities has also been reported in the literature [34]. However, it is worth noting that while surface plasmons facilitate light transmission through subwavelength metal gratings, they instead may present a detrimental effect when the slit periodicity aligns with multiples of the surface plasmon wavelength [20,35,36].

The majority of theoretical investigations on transmission through sub-wavelength slits in metal films assume the ideal scenario of perfect conductivity for the metal, which is not applicable at terahertz frequencies [37]. Consequently, at these frequencies, a more accurate description of the optical properties necessitates consideration of the actual properties of the metal [38]. Notably, as the frequency shifts to the terahertz range, the electromagnetic behavior of subwavelength slits acquires a lossy waveguide nature. Suckling J et al. demonstrated the imperative consideration of finite conductivity even in the low terahertz frequency regime [37]. Recently, J. E. Park et al. conducted a comprehensive investigation into the transmittance of sub-wavelength slits on Ag and Au plates. The study explored the influence of slit width and plate thickness across a frequency range spanning from 1.2 to 500 THz [39]. Their findings emphasize the dependency of transmittance on the electromagnetic properties of Ag and Au within the terahertz frequency range. Notably, the optical properties of the metal were carefully selected for specific frequencies in these investigations.

Though most of the mechanisms regarding EOT have been elucidated and reported between the late 90’s and the early 2010’s, arrays of slits continued to show significant interests both conceptually through recent reported studies in the diffraction limit [40], bound state in the continuum [41] topological [42], non-linear and tunable [43] metamaterials as well as practically across sensing [44], filtering [45] and wave guiding applications [46].

In this paper, we conduct a thorough examination of the optical behavior of an array of sub-wavelength metallic slits across a broad frequency spectrum, encompassing wavelengths ranging from the near-infrared to the visible. To account for both intraband and interband transitions occurring within the metal, we employ a model based on experimental data obtained from Johnson and Christy [47]. The primary objective of this analysis is to conceptualize structures that can efficiently interact with terahertz and optical waves at the sub-wavelength scale.

## 2. Structure Design and Theoretical Model

**Figure 1:**
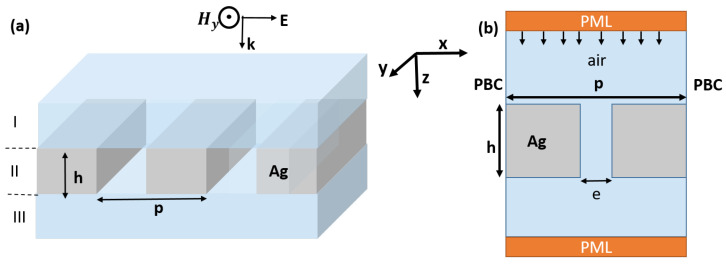
Schematic representation in 3D (**a**) and unit-cell (**b**) in 2D of silver plate structure featuring a sub-wavelength slit with a width *e*. The geometrical parameters employed in the design are as follows: the grating period *p* is equal to the plate thickness *h*. The width of slit is one-tenth of the grating periodicity ($e=\frac{p}{10}$).

In this study, we analyze the transmission behavior of an array of slits drilled within a metallic plate with dimensions smaller than the wavelength, in a wide frequency range. The structure under investigation is illustrated in Figure 1, and its specific geometrical parameters are detailed in the caption of the same figure. The width of each individual slit, denoted *e*, is set to one-tenth of the grating period (e=p/10). To thoroughly investigate the behavior of the structure over a broad frequency range, spanning from far-infrared to visible regions, we employ a scaling approach over the whole structure. From a numerical analysis perspective, we employ the finite element method (FEM) with a transverse magnetic TM-polarized wave launched from the top and propagating along the *y*-direction. The theoretical framework was chosen to appropriately align with the numerical results is the rigorous coupled wave analysis model (RCWA), which was first introduced by Moharam et al. in 1995 [48]. To address the periodicity along the *x*-direction, we implement a Floquet-Bloch condition for the elementary cell. The optical properties of the system, such as reflection, transmission, and absorption, are then assessed using two ports positioned along the *y*-direction—one at the top and another at the bottom. Additionally, to prevent reflections caused by impedance mismatch at the boundaries, we introduce perfectly matched layers (PML) along the propagation direction (*oy*). These PML effectively absorb spurious waves and ensure accurate results during the analysis.

**Figure 2:**
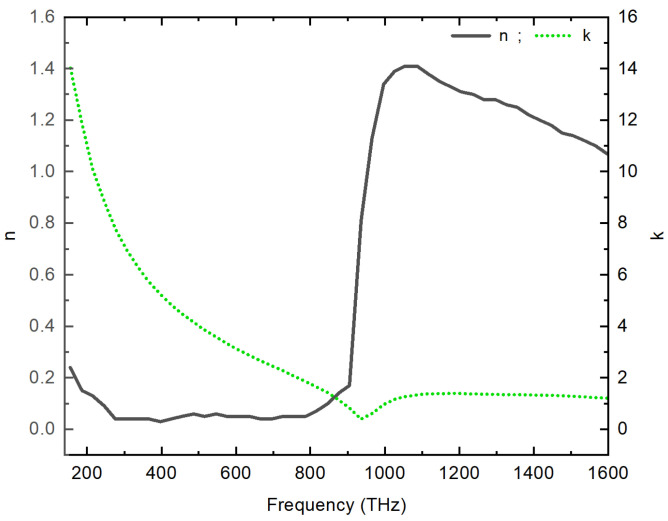
Dispersion Behavior of Silver: A graphical representation of the changes in the real part n(ω) and imaginary part k(ω) of the refractive index, using experimental data from Johnson and Christy.

The optical properties of silver (Ag) are described by a complex dielectric permittivity $\varepsilon_{m}\left(\omega \right)={\left(n\left(\omega \right)-jk\left(\omega \right)\right)}^{2}$, where n(ω) represents the real part, and k(ω) the imaginary part of silver’s refractive index at a given frequency ω. These properties exhibit frequency dependency, leading to dispersion effects. To analyze and understand how the optical properties of Ag change with frequency, we refer to experimental data presented by Johnson and Christy [47], as depicted in Figure 2.

### Theoretical Model: Rigorous Coupled Wave Analysis Model

To study a sub-wavelength metallic grating in air using the RCWA method, an initial step involves representing the relative permittivity within the grating through a Fourier series.

(1) $$\varepsilon \left(x\right)=\varepsilon_{0}+\sum_{m=1}\varepsilon_{m}e^{j\frac{2\pi }{p}m}$$

where $\varepsilon_{m}$ represents the Fourier harmonic of order *m* for the relative permittivity of the grating. For a grating consisting of alternating regions with refractive index $n_{a}$ and a complex index of the metal $n_{Ag}$, the Fourier harmonics are expressed as follows:

(2) $$\varepsilon_{0}=n_{a}^{2}\cdot f+n_{Ag}^{2}\left(1-f\right)$$

(3) $$\varepsilon_{m}=\left(n_{a}^{2}-n_{Ag}^{2}\right)\frac{\sin(\pi mf)}{\pi m}$$

where *f* denotes the fraction of the grating period occupied by air, and $\varepsilon_{0}$ represents the average value of the relative permittivity, which is determined using effective medium theory [49].

When an incident TM-polarized wave at an angle of incidence θ, interact with the structure, the magnetic fields of the incident, reflected, and transmitted waves are represented using a series of progressive plane waves. The expressions for the incident magnetic field in region I (see Figure 1a) is as follows:

(4) $$H_{in,y}=\exp\left(-j\left(k_{xI}\cdot x+k_{zI}\cdot z\right)\right)$$

Also, the magnetic fields of the reflected wave in region I and the transmitted wave in region III are defined by:

(5) $$\begin{array}{cc} \; & H_{I,y}=H_{in,y}+\sum_{i}R_{i}\exp\left(-j\left(k_{xi}\cdot x+k_{Izi}\cdot z\right)\right)\\ \; & H_{III,y}=\sum_{i}T_{i}\exp\left(-j\left(k_{xi}\cdot x+k_{IIIzi}\cdot \left(z-h\right)\right)\right) \end{array}$$

where,

(6) $$k_{xi}=k_{0}\left(n_{I}\sin\left(\theta \right)-i\frac{\lambda_{0}}{p}\right)$$

and

(7) $$\;k_{lzi}=\left\{\begin{array}{c} k_{0}{\left[n_{l}^{2}-{\left(\frac{k_{xi}}{k_{0}}\right)}^{2}\right]}^{1/2}\;\mathrm{si}\;k_{0}n_{l}>k_{xi}\\ -jk_{0}{\left[{\left(\frac{k_{xi}}{k_{0}}\right)}^{2}-n_{l}^{2}\right]}^{1/2}\;\mathrm{si}\;k_{0}n_{l}<k_{xi} \end{array}\right.$$

The index *l* can take the values of either I or III, corresponding to the reflected and transmitted regions, respectively. Additionally, $k_{0}$ represents the wave number in vacuum and it is defined as $k_{0}=\frac{2\pi }{\lambda }$.

The normalized amplitudes of the magnetic field for the *i*th wave diffracted forward and backward in region I and III are denoted by $R_{i}$ and $T_{i}$, respectively. The electric field in both regions can be easily determined using the Maxwell-Ampère equation.

(8) $$E=\left(\frac{-j}{\omega \varepsilon_{0}n^{2}}\right)\nabla \wedge H$$

Within the metal grating, the electric field E and magnetic field H are expressed using a generalized Fourier series.

(9) $$H_{gy}=\sum_{i}U_{yi}\left(z\right)\cdot \exp\left(-jk_{xi}x\right)$$

(10) $$E_{gx}=j{\left(\frac{\mu_{0}}{\varepsilon_{0}}\right)}^{1/2}\sum_{i}S_{xi}\left(z\right)\cdot \exp\left(-jk_{xi}x\right)$$

$U_{yi}$ and $S_{xi}$ are the normalized amplitudes of the $i_{mi}$th spatial harmonics for the $H_{gy}$ and $E_{gx}$ fields, respectively. Moreover, by applying Maxwell’s equations in conjunction with the developments of $H_{gy}$ and $E_{gx}$, we obtain the following system of differential equations:

(11) $$\left\{\begin{array}{c} \frac{\partial H_{gy}}{\partial z}=-j\omega \varepsilon_{0}\varepsilon \left(x\right)E_{gx}\\ \frac{\partial E_{gx}}{\partial z}=-j\omega \;\mu_{0}H_{gy}+\frac{\partial E_{gz}}{\partial x} \end{array}\right.$$

After substituting expressions Equations (1), (9) and (10) into the system of equations Equation (11), we derive a matrix equation, commonly referred to as the coupled wave equation.

(12) $$\left[\begin{array}{c} \frac{\partial U_{y}}{\partial \left(z^{\prime }\right)}\\ \frac{\partial S_{x}}{\partial \left(z^{\prime }\right)} \end{array}\right]=\left[\begin{array}{cc} 0 & A\\ B & 0 \end{array}\right]\left[\begin{array}{c} U_{y}\\ S_{x} \end{array}\right]$$

This matrix system can be simplified to the following form:

(13) $$\frac{\partial^{2}U_{y}}{\partial {\left(z^{\prime }\right)}^{2}}=\left[AB\right]\left[U_{y}\right]$$

where,

(14) $$z^{\prime }=k_{0}Z\;\;\mathrm{et}\;\;B=K_{x}A^{-1}K_{x}-I$$

With A denotes the matrix comprising the harmonic components of permittivity. $K_{x}$ represents a diagonal matrix, with its elements (i,i) equal to $k_{xi}/k_{0}$, where $k_{xi}$ is the wave number in the *x*-direction for the *i*th harmonic and $k_{0}$ is the wave number in vacuum. Additionally, *I* stands for the identity matrix.

By solving Equation (13), we can calculate the reflection and transmission coefficients, denoted as $R_{i}$ and $T_{i}$, respectively. These coefficients play a crucial role in determining the diffraction efficiency at order *i*.

(15) $$\left\{\begin{array}{c} DE_{ri}={\left|R_{i}\right|}^{2}Re\left(\frac{k_{I,xi}}{k_{0}n_{I}\cos\left(\theta \right)}\right)\\ DE_{ti}={\left|T_{i}\right|}^{2}Re\left(\frac{k_{II,xi}}{k_{0}n_{I}\cos\left(\theta \right)}\cdot \frac{n_{I}^{2}}{n_{II}^{2}}\right) \end{array}\right.$$

## 3. Results and Discussion

**Figure 3:**
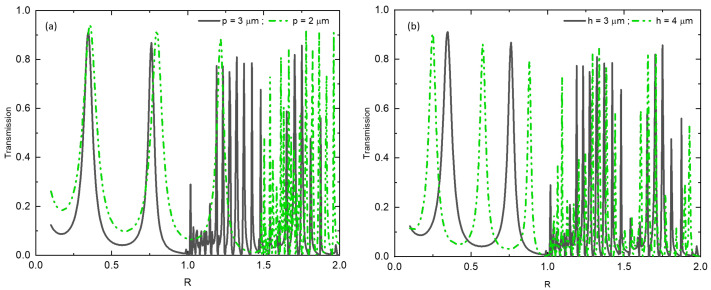
Transmission as a function of the reduced parameter R: (**a**) for two specific periodicity values of 2 µm and 3 µm, respectively. (**b**) for different grating thicknesses, *h* = 3 µm and *h* = 4 µm, with a fixed lateral periodicity of *p* = 3 µm, respectively.

Due to the wave nature of light, when it passes through apertures, the wave surface undergoes modifications, leading to a phenomenon known as diffraction. Extensive research has been conducted on diffraction, resulting in various models and approximations based on classical diffraction theory [50,51]. The diffraction limit is primarily determined by the lateral periodicity **p** of the metal grating, which serves as a controlling parameter for the diffraction process. In Figure 3a, we observe the light transmission as a function of the reduced parameter $R=\frac{p}{\lambda }$ for different values of the period *p*. The graph in Figure 3a clearly shows that, with a constant grating thickness of h=3μm, the diffraction limit is observed at R=1 for a grating with a periodicity of p=3μm. However, for a grating with a periodicity of p=2μm, the diffraction limit shifts to R=1.5. Interestingly, when varying the thickness of the metal grating while keeping the period constant at p=3μm (Figure 3b), the diffraction limit remains unchanged. However, increasing the thickness allows higher modes to be observed before the onset of diffraction.

**Figure 4:**
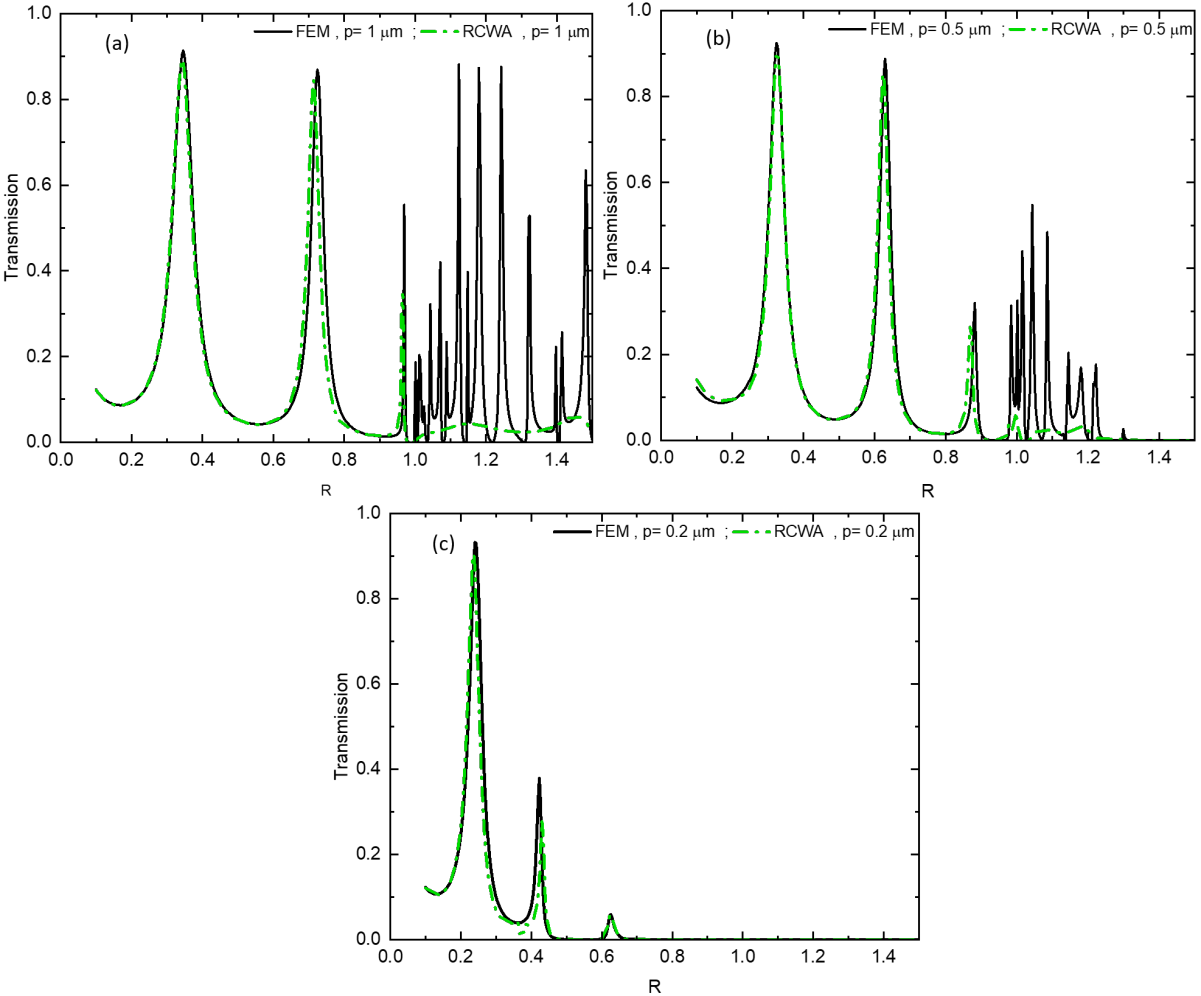
Comparison of transmission behavior as a function of the reduced parameter R between the FEM method (black line) and the RCWA theoretical model (green dashed line) for lateral periodicity values: (**a**) *p* = 1 µm, (**b**) *p* = 0.5 µm and (**c**) *p* = 0.2 µm.

To ensure the accuracy of our results, we performed a comparative analysis between the results derived from the finite element method and those obtained using the rigorous coupled wave analysis method. In Figure 4, a strong correlation is observed between the transmission curves obtained from the finite element method (black line) and the theoretical model based on the rigorous coupled wave analysis method (green dashed line), encompassing various regimes. Hereafter, we focus on the behavior of a metallic grating composed of sub-wavelength slits as it transitions from the regime under which the metal is deemed perfect, to the absorbing one. To achieve this, we scale up the square metal grating h=p to track the evolution of the two modes and the diffraction limit throughout the transition from perfect to absorbing behavior.

**Figure 5:**
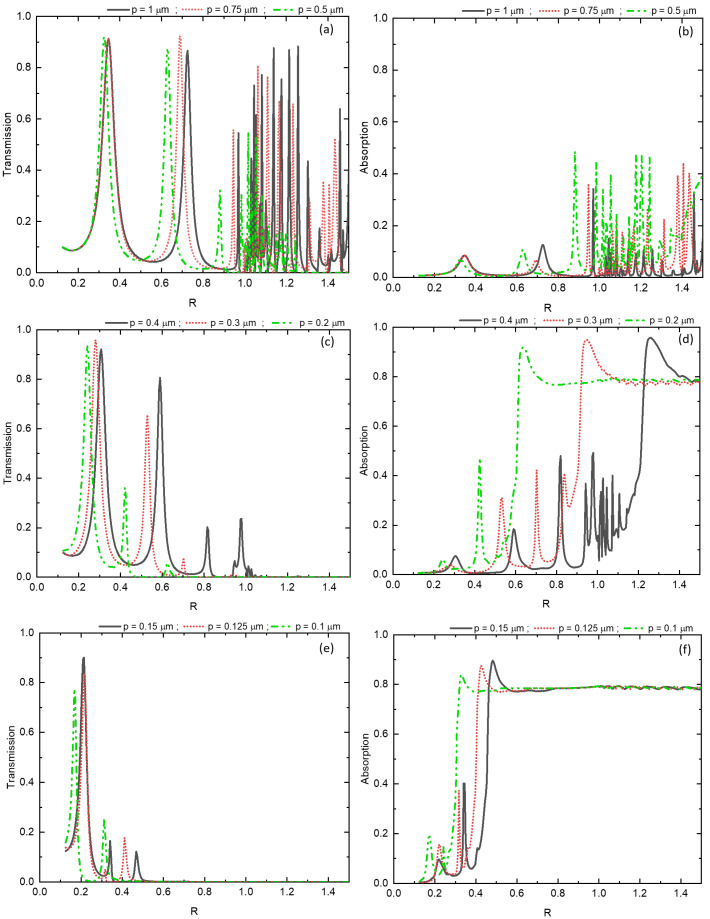
Transmission and absorption spectra of a sub-wavelength silver slit grating for different values of the period *p*.

Figure 5 illustrates the transmission and absorption spectra of a metal grating with sub-wavelength slits, as they vary with the reduced parameter R for different values of the square grating’s period *p*. For values of *p* greater than 0.5 µm Figure 5a,b, the metal behaves like a perfect conductor. The transmission peaks exhibit consistent intensity with a constant spacing between peaks, equal to half the wavelength, indicating different orders of the Fabry-Perot resonance modes. As the period *p* decreases below 0.5 µm Figure 5c,e, the transmission peak of the λ mode gradually diminishes. For instance, the transmission coefficient decreases from T=0.92 for p=0.75μm to T=0.36 for p=0.2μm.

The observed behavior is attributed to the interband transitions of silver, which become more pronounced as p decreases. As a result, a portion of the energy is absorbed by the metal Figure 5d,f, leading to an attenuation of the transmission peaks in the near-infrared and visible ranges. Additionally, there is a noticeable shift in the transmission peaks towards lower frequencies as *p* decreases. This shift is particularly pronounced at the resonance peaks; for instance, at p=0.75μm, the peak is located at R=0.688, while for p=0.2μm, it shifts to R=0.42. This highlights a non-linear behavior of the resonance frequencies concerning the periodicity of the sub-wavelength grating.

**Figure 6:**
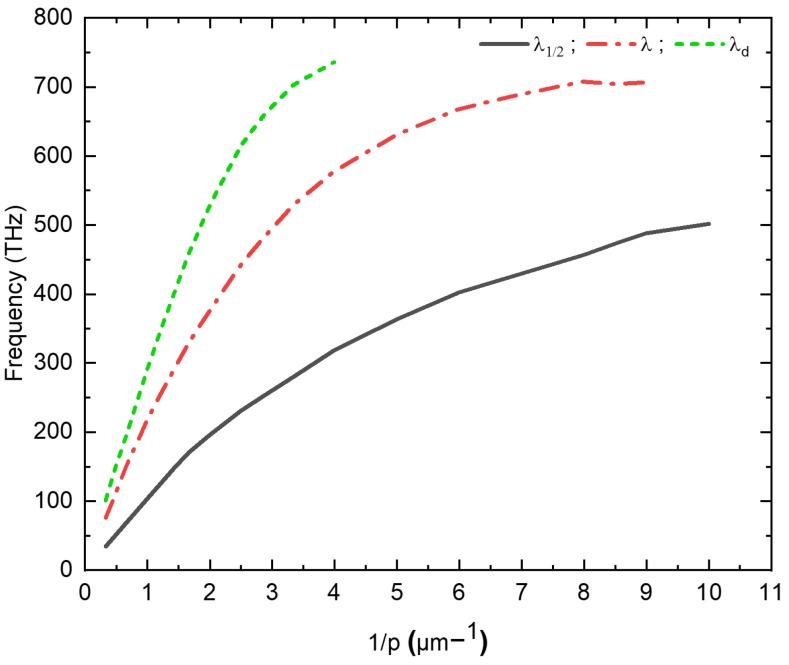
The dependency of resonance frequency peaks $\lambda_{1/2}$, λ, and $\lambda_{d}$ diffraction limit on the inverse of the period (*p*).

To elucidate the evolution of the resonance peaks $\lambda_{1/2}$ which means the first effective Fabry-Perot mode and λ, along with the diffraction limit, the resonance frequencies corresponding to each mode were graphically depicted against the reciprocal of the lateral period (see Figure 6). This figure predicts two distinct behaviors of these modes based on the geometric parameters of the nanostructure. Evidently, when the periodicity *p* attains or exceeds the threshold of 0.5μm (p≥0.5μm), the resonant frequencies pertaining to the three modes exhibit a linear evolution in correspondence with the inverse of the periodicity (1/p). The origin of this resonant transmission emanates from the harmonious coupling between the TEM-guided mode and the resonant modes of the opened cavities, or slots. It is noteworthy that only the metallic walls of these slots take an active part in this phenomenon. However, a notable deviation from linearity becomes evident when *p* falls below 0.5μm (p<0.5μm), as delineated by curve in Figure 6.

**Figure 7:**
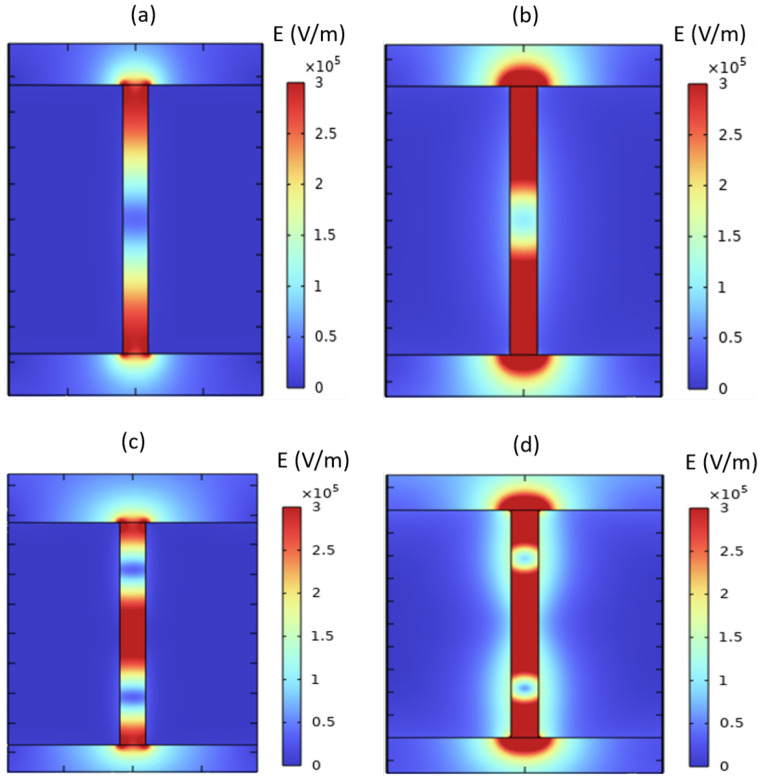
Electric field norm maps for both $\lambda_{1/2}$, and λ resonant modes at two different periodicities of p=0.2μm (**b**,**d**) and p=0.75μm (**a**,**c**).

Within this terahertz frequency range, the optical characteristics of the metallic material assume a pivotal role, causing considerable attenuation of the resonance modes due to an absorbing regime. Consequently, a noteworthy proportion of energy undergoes absorption by the metal, thereby contributing to the significant reduction in transmission through the sub-wavelength slit gratings. Moreover, it should be emphasized that the utilization of nanoscale metal slots introduces a propensity for generating localized resonances, wherein electromagnetic waves become entrapped and magnified at specific frequencies, leading to pronounced transmission attenuation. This intriguing behavior is clearly represented through field maps in Figure 7, illustrating the precise localization of electromagnetic energy. At a periodicity of p=0.75μm, the resonant modes $\lambda_{1/2}$ Figure 7a and λ Figure 7c exhibit no attenuation, displaying a pronounced localization of energy within the slits of the grating. This localization phenomenon confirms the behavior characteristic of a perfect conductor for the metallic material. Conversely, when *p* decreases to 0.2μm, the $\lambda_{1/2}$ Figure 7b mode remains unattenuated, allowing energy transmission through the slits. Nevertheless, for *p* values below 0.2μm, this particular peak gradually experiences attenuation.

In contrast, the mode λ (Figure 7d) exhibits a distinct behavior, wherein a portion of its energy disperses into the metallic medium, penetrating to a depth dictated by the skin effect. This skin effect depth is proportionally dependent on the reciprocal of the product encompassing the real and imaginary components of the refractive index. Consequently, as the dimensions of the grating decrease below p=0.1μm, the λ mode undergoes complete attenuation. Furthermore, it is pertinent to note the emergence of a transmission mode specific to the metallic material, observed at the frequency of 933THz. Remarkably, the amplitude of this mode escalates with a reduction in periodicity, augmenting its significance as the dimensions of the grating decrease.

**Figure 8:**
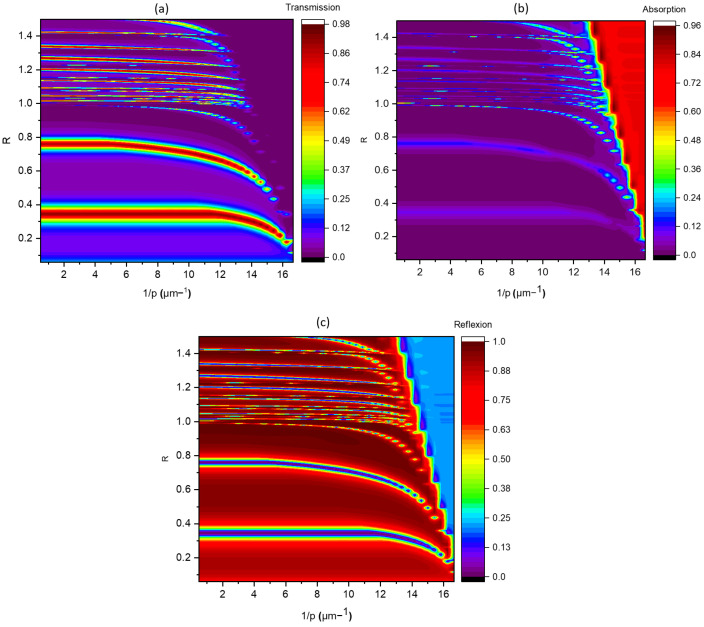
Transmission (**a**), reflection (**c**) and absorption (**b**) map as a function of the parameter R and the inverse of the period.

To comprehensively trace the intensity evolution of the three aforementioned modes during the scaling of the subwavelength grating. A detailed depiction of transmission, reflection, and absorption behaviors has been demonstrated in the maps in Figure 8, unveiling the intricate dependencies on the reduced parameter R and the reciprocal of the period (1/p). Particularly, when the value of 1/p is less than or equal to 2, the $\lambda_{1/2}$ and λ modes consistently maintain a nearly identical transmission coefficient of about T=0.96. Moreover, an unvarying spectral interval of approximately ∆R≈0.4 separates these modes. Additionally, it is noteworthy that the diffraction limit distinctly converges at R=1.

In the regime where $\frac{1}{p}\geq 2$, a notable decline in the intensity transmitted by the λ mode is observed as the periodicity *p* diminishes. Concurrently, the spectral interval between successive peaks also exhibits a progressive reduction. These observations unequivocally indicate the pronounced impact of the dispersive nature of the metallic material on both the peak intensity and their respective positions. Moreover, this effect becomes more pronounced as the mode order increases. As the dimensions of the sub-wavelength grating are successively reduced, a discernible trend towards increased selectivity emerges, indicative of the grating’s heightened sensitivity to variations in the mode frequencies and intensities.

## 4. Conclusions

In conclusion, the investigation of sub-wavelength metallic slit gratings presents a captivating realm of research encompassing an extensive frequency spectrum. In this particular study, our focus was on exploring the behavior of a metallic silver slit grating (h=p), scaled across radio frequencies to optical frequencies. At lower frequencies, the metallic material exhibits a perfect conductor-like behavior concerning the Fabry-Perot resonance modes within the slits. However, as we shift into the terahertz frequency range, the optical properties of the metal assume paramount significance, leading to a discernible attenuation of the resonance modes. Notably, solely the $\lambda_{1/2}$ mode persists despite the metal’s absorption characteristics within the examined range. These gratings offer a myriad of possibilities for the design and development of diverse optoelectronic devices, encompassing sensors, filters, and antennas. As fabrication and characterization techniques for these gratings continue to advance, it becomes essential to delve into further research endeavors to unlock their full potential. This potential encompasses enhancing the sensitivity of optical sensors, devising miniaturized optical devices, and propelling the advancement of high-speed optical communication technologies.

## Data Availability

The data presented in this study are available on request from the corresponding author.
